# Patterns of prostate recurrence after focal salvage prostate brachytherapy for radiorecurrent prostate cancer

**DOI:** 10.1016/j.ctro.2025.101043

**Published:** 2025-09-04

**Authors:** Alba Domínguez Domínguez, Niels den Haan, Jan Wiersma, Josephina C.C. Koppes, Karel A. Hinnen, Bradley R. Pieters

**Affiliations:** a Department of Radiation Oncology, Amsterdam UMC location University of Amsterdam, Amsterdam, The Netherlands; b Cancer Center Amsterdam, Cancer Treatment and Quality of Life, Amsterdam, The Netherlands; c Department of Radiology and Nuclear Medicine, Amsterdam UMC location Vrije Universiteit, Amsterdam, The Netherlands; d Cancer Center Amsterdam, Imaging and Biomarkers, Amsterdam, The Netherlands

**Keywords:** Salvage prostate brachytherapy, Pattern of recurrences, Local recurrences, Clinical target volume

## Abstract

**Background and purpose:**

Focal high-dose-rate (HDR) salvage brachytherapy has emerged as a treatment for radiorecurrent prostate cancer. This study aims to evaluate patterns of recurrence after focal salvage brachytherapy and to assess the adequacy of current treatment margins.

**Materials and methods:**

Between March 2015 and December 2021, 39 patients with radiorecurrent prostate cancer underwent focal HDR brachytherapy. All patients had biopsy-confirmed local recurrence and were staged using Choline- or PSMA-PET/CT and multiparametric MRI. A 5 mm margin around the GTV was applied to define the CTV. Post-treatment recurrences were analyzed using rigid image registration of PET/CT and MRI to assess spatial relationships among the initial recurrence (Rec1), the recurrence following salvage brachytherapy (Rec2), and the brachytherapy dose distribution. The recurrences were categorized into infield, marginal, and outfield based on overlap of relapse with the treated CTV and based on dose received on the site of the relapse. Additionally, spatial analysis measured minimal distances between Rec1 and Rec2.

**Results:**

Nineteen of 39 patients experienced clinical recurrence, with 12 exhibiting 25 local lesions. Based on spatial overlap, 20 % of Rec2 lesions were infield, 28 % marginal, and 52 % outfield. Dose-based classification indicated 52 % infield, 8 % marginal, and 40 % outfield recurrence. The median distance between Rec1 and Rec2 in outfield cases was 11.9–13.4 mm.

**Conclusion:**

A substantial proportion of local recurrences after focal salvage brachytherapy occur outside the treated volume. Current 5 mm margins may be insufficient.

## Highlights

• 40–52 % of prostate recurrences after focal salvage brachytherapy are classified as outfield recurrences.
• The outfield recurrences occur at a minimal median distance of 11.9–13.4 mm from the treated GTV with salvage brachytherapy.
• A CTV margin of 5 mm in focal salvage brachytherapy may be inadequate for optimal disease control.

## 1 Introduction

Salvage brachytherapy for prostate cancer recurrence after previous radiotherapy is an emerging treatment modality, with nuclear imaging techniques, such as PSMA-PET, enabling the identification of local relapses at an early stage and the potential for local treatment to cure patients [1].

As with any reirradiation treatment salvage brachytherapy may lead to side effects including bleeding, stenosis, ulcer, necrosis or fistula. A number of publications concerning salvage brachytherapy for radiorecurrent prostate cancer have demonstrated the efficacy of this modality [[2], [3], [4], [5]]. The results of HDR salvage brachytherapy are summarized in a systematic review showing a 5-year recurrence-free survival rate of 60 %. Reported severe genito-urinary (GU) and gastrointestinal (GI) toxicity are 8 % and 0 %, respectively. Of the 16 HDR studies in this systematic review 85 % used whole gland salvage brachytherapy as treatment [1].

Less common is the use of focal brachytherapy in this respect. An advantage of focal treatment is that the treated volume can be reduced, which may consequently result in less toxicity. The toxicity levels reported in a limited number of studies are low, grade 3 GU toxicity between 0 % and 10 % and grade 2 GI toxicity between 0 % and 8 % [2,[5], [6], [7]]]. Interestingly, the relapse free survival after partial gland brachytherapy seems not to be inferior to whole gland brachytherapy [1].

Since 2015 the Amsterdam UMC has performed focal salvage HDR brachytherapy in cases of radiorecurrent prostate cancer, with these patients having received primary treatment of solely external beam radiotherapy (EBRT), EBRT combined with pulsed-dose rate (PDR) brachytherapy, or I125 implantation.

Because we were still seeing local prostate recurences after salvage brachytherapy we initiated a study to assess the pattern of local recurrences. The aim of this study is to assess the adequacy of the focal treatment volume and to provide recommendations for margins in cases of focal salvage brachytherapy.

## 2 Materials and methods

### 2.1 Patient selection

In the period March 2015 until December 2021 a total of 39 patients diagnosed with radiorecurrent prostate cancer were treated with focal salvage brachytherapy. Patients were diagnosed because of an increasing PSA after primary treatment. Patients obtained a choline-PET/CT scan and in later years PSMA-PET/CT for diagnosis and to exclude distant metastasis. Three patients with a solitary lymph node metastasis were accepted for treatment. A multiparametric MRI (mpMRI) was done to assess the exact location of the recurrent disease. All patients obtained at least systematic prostate biopsies to confirm focal disease.

The minimum interval between primary and salvage treatment must be a minimum of two years and the PSA doubling time must exceed six months.

The protocol was approved by the Medical Ethics Review committee of the Amsterdam UMC (number of approval 2025.0427).

### 2.2 Treatment

All patients underwent transrectal ultrasound-guided transperineal implantations in lithotomy position with the aid of the Martinez Prostate Template (Elekta, Stockholm, Sweden). The positions at which to implant the needles were decided on cognitive fusion of the PET-scan and mpMRI and histology findings in order to cover the recurrent tumor with a 5 mm margin.

After implantation, a T2-weighted MRI with the needles in situ was conducted for definitive brachytherapy treatment planning.

The delineation of the target volume and treatment planning were done using Oncentra® (Elekta, Stockholm, Sweden). The gross target volume (GTV) was delineated on the MRI, with a 5 mm margin applied for the clinical target volume (CTV), limited to 3 mm beyond the prostate or seminal vesicle contours. In cases of discrepancies in tumor location as determined by the pretreatment PET-scan, mpMRI and biopsies, a larger CTV margin was used.

The applied dose was 3 fractions of 10 Gy within a single implantation except for one patient (Table 1), with the treatment planning aims being V_100__PTV ≥ 95 %, D_90_ ≥ 10 Gy/fraction, D_2cm_^3^_Rectum ≤ 80 % (8 Gy/fraction), and D_0.1cm_^3^_Urethra ≤ 120 % (12 Gy/fraction).

**Table 1:** Patient and tumor characteristics at primary and salvage treatment.

**Patient characteristics**	Primary treatment	Salvage treatment
Age	Median 64.4 years (SD 6.2)	Median 74.3 years (SD 5.2)
T classification		
T1	7	17
T2	23	8
T3a	8	2
T3b	0	10
Unknown	1	2
N classification		
N0	38	36
N1	0	3
Unknown	1	0
ISUP classification		
ISUP 1	21	2
ISUP 2	10	6
ISUP 3	2	9
ISUP 4	1	11
ISUP 5	1	7
Unknown	4	4
PSA	Median 12.0 ng/mL (range 4.5–98.3)	Median 4.6 ng/mL Range (0.7–19.5)
Diagnostics		
Choline PET		17
PSMA PET		22
MRI abdomen		39
**Treatment characteristics**		
Low dose EBRT < 76 Gy	13	
Dose escalated EBRT ≥ 76 Gy	12	
I125	12	
EBRT + PDR boost	2	
Salvage brachytherapy dose		
10 × 3.0 Gy		38
4 × 8.5 Gy		1
Elective nodal irradiation	Yes 3	Yes 0
	No 36	No 39
SBRT lymph nodes	Yes 0	Yes 2
	No 39	No 37
Lymph node dissection	Yes 8	Yes 0
	No 31	No 39
Androgen deprivation therapy	Yes 13	Yes 4
	No 26	No 35

Legend: ISUP: The International Society of Urological Pathology, EBRT: External beam radiotherapy, PDR: Pulsed-dose rate, SBRT: Stereotactic Body Radiotherapy.

The first fraction was administered on the day of the implantation, with the subsequent two fractions the day after, with a minimal six hours interval between each. On the second day a CT-scan was performed to ascertain for needle displacement, and if necessary, the needles were repositioned and the treatment plan revised.

### 2.3 PSMA PET-CT protocol

In accordance with the local protocol PSMA PET was combined with either a non– contrast enhanced low dose CT scan or a diagnostic CT scan with or without intravenous contrast. Three various PSMA radiotracers were used, including [18F]DCFPyL, [68 Ga]Ga- PSMA-11 or [18F]-Choline PSMA PET-CT.

### 2.4 Assessment of PSMA PET-CT

The PSMA PET-CT was assessed independently by one or two experienced nuclear medicine physicians. A positive scan was defined as the presence of at least adequate (more than the background and bloodpool) PSMA uptake in the prostate region.

The lesions were marked using the build in tool of Hermes (HERMIA, Stockholm, Sweden). Hermes is used in nuclear medicine to improve lesion identification by combining anatomical details from CT scans with metabolic data from PET imaging.

### 2.5 Contouring of radiorecurrent recurrence and recurrence after salvage brachytherapy

All available images, the contoured GTV and CTV, and dose plans were imported into Velocity (Varian Medical Systems, Palo Alto, USA) for further analysis.

**Fig. 1:**
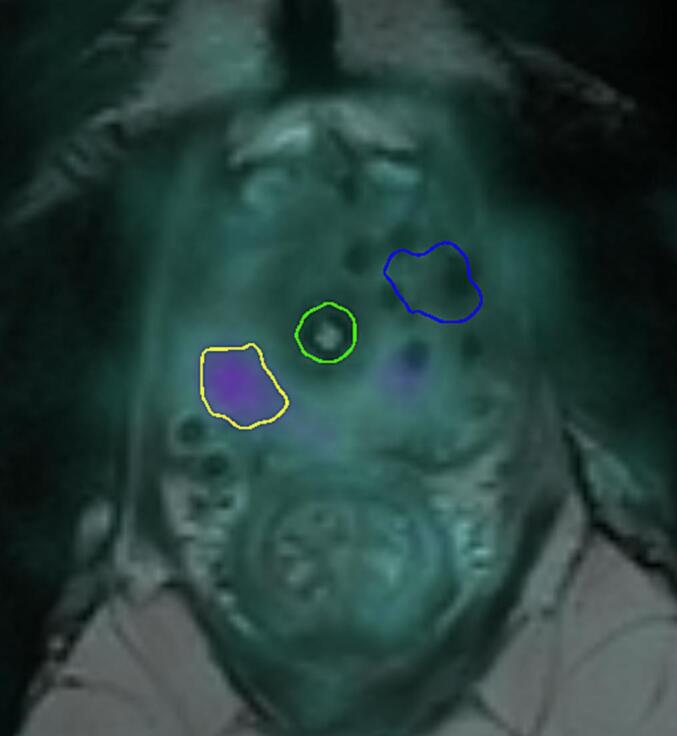
Contoured Rec1 in blue and Rec2 in yellow after registration of the PSMA scan at salvage treatment with PSMA scan at second recurrence and with the brachytherapy T2-MRI with implanted needles.

The initial radiorecurrent disease (Rec1 or first recurrence) was contoured on the Choline-PET/CT or PSMA- PET/CT scan prior to salvage brachytherapy. The subsequent relapse after focal salvage brachytherapy (Rec2 or second recurrence) was contoured on the PSMA-PET/CT after treatment (Fig. 1). An MRI was used for contouring Rec1 in one patient because a PET/CT scan was not available.

### 2.6 Standardization of PET-CT contouring

In consultation with a nuclear medicine physician, a PET-CT standard was established. Patients were contoured on the attenuation-corrected (AC) sequence of the PET scan with an SUVbw range of 0–7. Any conflicts of uncertainties were resolved by discussion between the nuclear medicine physician and radiation oncologist.

### 2.7 Registration

In order to determine the spatial relation of Rec1 to Rec2, the PET/CT at initial radiorecurrent disease is rigidly registered (translations/rotations only) to the PET/CT at relapse. The Rec1 delineation is copied/mapped to the PET/CT at relapse by applying this registration. The spatial relation of Rec2, CTV and planned brachytherapy dose is likewise determined but now by rigidly registering the PET/CT at relapse to the brachytherapy treatment planning MRI. This registration is applied to copy/map CTV and dose to the PET/CT at relapse. This approach enables copying/mapping Rec1, CTV, administered brachytherapy dose and Rec2 on the PET/CT at relapse and reporting on geometrical and dose overlap. In case of prostate implanted gold-markers or other fiducials these were used to determine the registration.

### 2.8 Study design

#### 2.8.1 The overlap of Rec2 with the CTV of Rec1

**Fig. 2:**
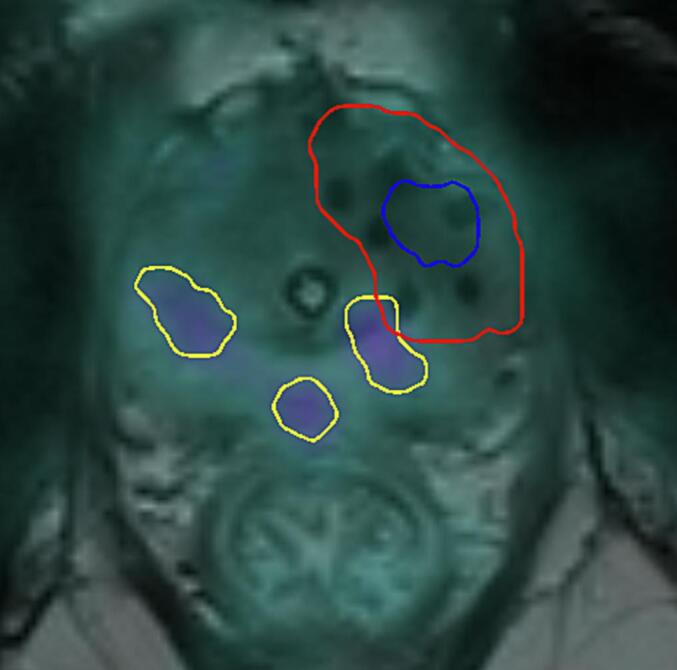
Rec1 in blue with PTV in red. Three recurrent lesions after salvage brachytherapy in yellow. Two of these lesions were categorized as outfield based on the ovelap definition and one as marginal.

The imported treatment planning MRI dataset contained the CTV as the actual target volume for treatment planning. The overlap of Rec2 with CTV was calculated and recurrences based on this overlap were categorized as infield (≥30 % overlap), marginal (<30 % and >0 % overlap), and outfield (0 % overlap) (Fig. 2 and Suppl. Fig. 1).

#### 2.8.2 Relation of Rec1 and Rec2 with regard to the dose

**Fig. 3:**
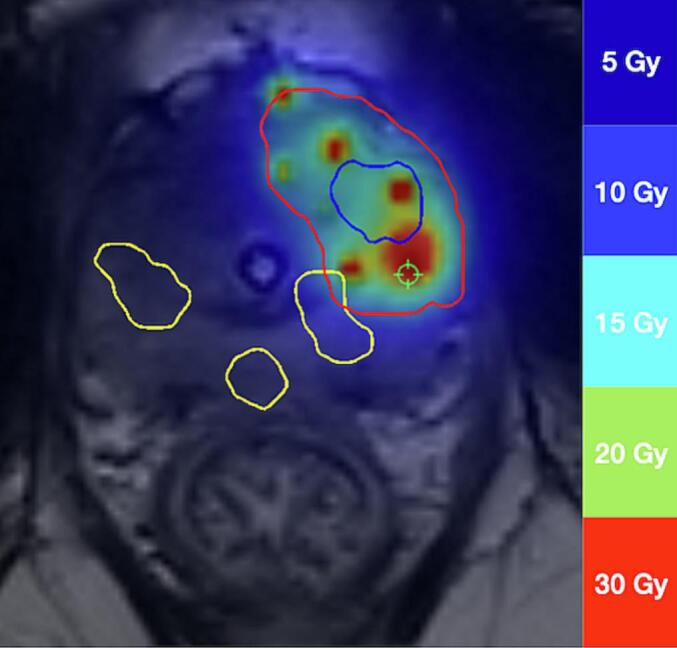
Dose color wash of brachytherapy treatment. Rec1 in blue with PTV in red. Three recurrent lesions after salvage brachytherapy in yellow. Two of these lesions were categorized as outfield based on the dose definition and one as marginal. Dose numbers are doses per fraction.

The imported treatment planning MRI dataset contained information on dose. We calculated the maximum dose received by the site of Rec2 to classify recurrences based on dose. The higher the dose at the site, the closer Rec2 should be to the implantation site. The D_0.1cm_^3^ on the Rec2 was used to categorize recurrences into infield (≥ 25 Gy), marginal (< 25 Gy and ≥15 Gy), and outfield (< 15 Gy) (Fig. 3 and Suppl. Fig.1).

#### 2.8.3 Relation Rec1 and Rec2 on distance

The minimal distance between Rec1 and Rec2 was calculated to investigate the distance at which Rec2 occurs relative to Rec1. These distances were distributed according to the definitions of infield, marginal, and outfield.

#### 2.8.4 Statistics

The recurrence patterns observed at various sites were expressed as proportions with a 95 % confidence interval.The number of recurrences according to the three definitions of recurrences was described using descriptive statistics.

## 3 Results

A total of 39 patients who were treated for radiorecurrent prostate cancer were included. The patient and tumor characteristics at the time of primary and salvage treatment are summarized in Table 1.

It is noteworthy the number of second recurrences in the seminal vesicles (10 T3b), while no patient at primary treatment had seminal vesicle involvement. The median time to clinical recurrence was 24 months (range 6–87 months), and the median follow-up duration was 43 months (range 6–96 months).

The only patient who received 4 x 8.5 Gy did not recur locally, and thus this adapted dose was not considered in the further analysis.

In the 39 patients with focal salvage brachytherapy, 19 developed any clinical recurrence after focal salvage brachytherapy. Of these 19 recurrences, twelve (30.8 %; 16.3–45.3) occurred only in the prostate or seminal vesicle, 4 (10.3 %; 0.7–19.8) in the pelvic lymph nodes with or without a local recurrence, and 3 (7.7 %; 0–16.1) developed distant metastases nodes with or without a local recurrence (Table 2).

**Table 2:** Outcome of patients treated with salvage prostate brachytherapy.

Interval time	Median 9.0 months (range 2.0–21.8)
PSA doubling time	Median 43.0 months (range 6–96)
Recurrence site	None 20
	Prostate and/or seminal vesicles 12
	Pelvic lymph node and seminal vesicle 1 Pelvic lymph nodes 3 Distant metastasis and seminal vesicle 1
	Distant metastasis 2
Time to clinical recurrence	Median 24 months (range 6–87)
Follow up time	Median 43 months (range 6–96)

Legend: PSA: Prostate specific antigen.

We included in our investigations of pattern of local recurrence the 12 patients with only local recurrence that potentially could have been cured if a second relapse was prevented.

The imaging modalities used for the contouring of the first and second local recurrences of the 12 patients with local relapse after focal salvage brachytherapy are outlined in Table 3. Contouring of Rec1 was done with choline-PET in 6 patients, PSMA-PET in 8 patients, and MRI in one patient. For the contouring of Rec2 only PSMA-PET was utilized.

**Table 3:** Imaging techniques used for contouring the local recurrences.

Imaging techniques	Rec1	Rec2
Choline PET	6	0
PSMA PET	5	12
MRI	1	0

In these 12 patients, 25 separate local recurrences (prostate or seminal vesicles) were identified. Five patients had one recurrence, four had two, one with three, one with four, and one with five.

In accordance with the overlap criteria, 5 (20 %) lesions correspond to infield recurrence, 7 (28 %) a marginal recurrence, and 13 (52 %) as a true outfield recurrence. The minimal distance of Rec1 to Rec 2 was measured as a median 5.2 mm in case of infield recurrence, 1.0 mm in case of marginal recurrence, and 11.9 mm in case of outfield recurrence (Table 4).

**Table 4:** Recurrence classification based on overlap and dose definition.

Definition		Median minimal distance Rec1-Rec2 (mm)	Range (mm)
Infield Overlap ≥ 30 %	5	5.21	0.00–7.56
Marginal Overlap > 0 %, <30 %	7	1.04	0.00–16.50
Outfield Overlap = 0 %	13	11.91	3.30–28.39
Infield ≥ 25 Gy	13	3.30	0.00–16.50
Marginal < 25 Gy ≥ 15 Gy	2	4.30	3.30–5.24
Outfield < 15 Gy	10	13.36	6.25–28.39

In case of dose definition, 13 (52 %) lesions correspond to infield recurrence, 2 (8 %) a marginal recurrence, and 10 (40 %) an outfield recurrence. The median minimal distance of Rec1 to Rec2 was measured as 3.3 mm in case of infield recurrence, 4.3 mm in case of marginal recurrence, and 13.4 mm in case of outfield recurrence (Table 4).

## 4 Discussion

This study represents to our knowledge the first analysis of patterns of local recurrence after focal salvage prostate HDR brachytherapy. Most previous studies have focused on biochemical outcomes rather than on the distribution of recurrences between first and second recurrences with dose and overlap.

In this study, 69 % of the treated relapses after primary treatment were located in the prostate and in 26 % in the seminal vesicles. Focal salvage brachytherapy was chosen with the objective of minimising treatment-related morbidity. The employment of modern imaging techniques, such as mpMRI and PSMA/PET, enabled the delineation of the treated area to be confined to the visible tumour on the imaging modalitie. Limited CTV margins of 3–5 mm around the visible tumor were used.

Our study showed that 40 % to 52 % of the recurrent lesions following focal salvage brachytherapy were outfield recurrences dependent on the classification used. This observation calls into question the necessity of the tight margins that are used as salvage treatment.

Venkatasulu et al. investigated the sensitivity of mpMRI and PSMA PET/CT (18F-Fluciclovine and 18F-DCFPyl) in detecting intraprostatic recurrences and correlation with histology findings in a phase I/II prospective trial [8]. The study revealed that both imaging modalities exhibited high sensitivity, with mpMRI demonstrating a sensitivity of 91.8 % and PSMA PET/CT showing 85.5 %. However, in 70.5 % and 73.8 % of cases with mpMRI and PSMA PET/CT, respectively, the tumor was found beyond the identified lesions on imaging. This study underscores the necessity for carefull attention to be paid in defining margins for treatment. In accordance with the findings of this trial, our study identified recurrences that were located beyond the site of the treated lesion. Despite the fact that a large number of recurrences based on the dose definition were infield (52 %), in only 20 % of lesions had an overlap with the CTV of more than 30 %, suggesting that the margins were too limited. When outfield recurrences are considered, it is clear that wider margins from the GTV need to be used and consequently the 5 mm margin used appeared to be inadequate. The median distance between the treated lesion and the relapsed outfield lesion post-salvage brachytherapy was 11.9–13.4 mm.

In the present study, the overall crude rate of recurrence rate after salvage brachytherapy was 49 %, with 31 % of cases exhibiting only a local recurrence in the prostate or seminal vesicles. These outcomes are consistent with those observed in other studies examining the results of focal prostate salvage brachytherapy.

In the study by Chitmanee et al., fifty patients were analysed with a median follow-up time of 21 months [2]. In this study, the CTV encompassed the region of the positive biospies with information of mpMRI and choline-PET, with a 3 mm margin for the planning target volume (PTV). A single 19 Gy dose was applied resulting in a 3-year biochemical relapse free survival of 46 %.

Slevin et al. also applied a single 19 Gy dose in a cohort of 43 patients [5]. In this study, the GTV was defined as the area of template-guided positive biopsies with information from mpMRI and either 18F-Choline or 18F-Flucliclovine PET/CT. The 3-year biochemical progression free survival was 41.8 % after a median follow-up time of 26 months.

A study with a more favorable outcome was reported by Murgic et al. In this study which included fifteen patients, a relative extensive area was defined as the CTV, encompassing the quadrant of the of the location of the tumor on mpMRI and a minimum of two-thirds of the prostate volume in the superior-inferior direction [3]. The 3-year biochemical relapse free survival was found to be 61 % with a dose of 2 fractions of 13.5 Gy.

The largest study on focal prostate brachytherapy is reported by Rasing et al. In this study, 175 patients were treated with a single 19 Gy dose of HDR brachytherapy [9]. The GTV was defined as the visible lesion on mpMRI and PSMA-PET/CT. A 5 mm margin was used from GTV to CTV. After a median follow-up period of 36 months, the 3-year biochemical relapse free survival was 43 % while the 3-year local recurrence free survival was 51 %. Notably, in this study few outfield recurrences were observed in the prostate, resulting in a 3-year prostate outfield local recurrence free survival of 92 %. A notable distinction between the present study and that of Rasing is the methodology employed; the former utilised a per-patient analysis, whereas in our study a per-lesion analysis was done. This discrepancy in analytical approach limits the direct comparability between the two studies.

The question of whether there is a preference between whole gland and focal salvage brachytherapy when considering both local control and toxicity remains unresolved [10,11]. A narrative systematic review of 20 studies revealed that whole gland 3-year biochemical failure free survival ranged from 48 % to 94 %, while in focal therapy, it ranged from 42 to 71 % [12]. This systematic review suggests that the two treatments have similar outcomes. However, a lower toxicity was observed in focal brachytherapy, with grade 3 or more GU and GI toxicity recorded at 3 % and 0 %, respectively, in contrast to the 12 % and 4 % observed in whole gland brachytherapy. This finding is in contrast to the results of another *meta*-analysis, which reported an unadjusted hazard ratio of 0.6 for whole gland treatment compared to focal brachytherapy, without a comparison of toxicity outcomes [13]. The optimal approach to ascertain the comparative value of these treatment modalities would be a randomized study.

In this study, PSMA-PET was used to contour Rec1 and Rec2. The rationale for employing only PSMA-PET was because this was the imaging modality that was available both prior to and following salvage brachytherapy. In the event of a recurrence after salvage brachytherapy, PSMA-PET/CT was mostly the only imaging modality because performing an additional MRI had no consequence. The patients were typically treated with androgen deprivation therapy, or in cases where PSA levels were low, underwent active monitoring.

In a prospective trial of patients with biochemical relapse standard diagnostic imaging (contrast-enhanced CT, bone scintigraphy, and mpMRI) was compared to 18F-DCFPyl PET/CT. The study revealed that intraprostatic recurrences were identified in 48 % of cases through standard diagnostic imaging, as compared to 68 % through 18F-DCFPyl PET/CT [14]. The study demonstrated a 95 % agreement among independent observers, underscoring the efficacy of PSMA PET/CT as a reliable imaging modality for localizing intraprostatic recurrences.

The predictive ability of PSMA-PET to identify malignant lesions in the prostate is high. A systematic review of 68 Ga-PSMA PET correlated to histology found a sensitivity of 75 % and a specificity of 99 % [15].

This study has several limitations. Firstly, the retrospective design precludes the testing of the association of different margins with local recurrence. We used rigid registration to assess the relation between lesions at two different time points. The use of androgen deprivation therapy and the fact that patients were treated with radiation therapy may have influenced the volume and shape of the prostate.The imprecision of the registration process must be considered when interpreting the results. Furthermore, the study was unable to ascertain the primary tumour location at the initial treatment stage. The majority of patients had their primary treatment in the early 2000s, during a period when there was no routine use of MRI, PSMA, and prostate biopsies were not usually categorized in prostate sections. Consequently, it cannot be excluded that for some patients, the primary tumour was not included in the salvage treatment area, which could potentially lead to a risk area for recurrence. Finally, it is acknowledged that long-term follow-up may lead to revisions of the results if further recurrences were to occur.

## 5 Conclusion

This study provides a detailed analysis of spatial recurrence patterns after focal salvage HDR brachytherapy for radiorecurrent prostate cancer. The findings demonstrate that a significant proportion of local recurrences occur outside the initially targeted treatment volume, despite the use of advanced imaging modalities such as mpMRI and PSMA-PET/CT. The high rate of outfield recurrences suggests that the currently applied 5 mm margin around the GTV may be inadequate for optimal disease control.

## Declaration of competing interest

The authors declare that they have no known competing financial interests or personal relationships that could have appeared to influence the work reported in this paper.
