# Supplementary figures and images for: Patterns of prostate recurrence after focal salvage prostate brachytherapy for radiorecurrent prostate cancer
Source: Clin Transl Radiat Oncol. 2025 Sep 4;56:101043. doi: 10.1016/j.ctro.2025.101043 (PMC12797306; doi:10.1016/j.ctro.2025.101043)

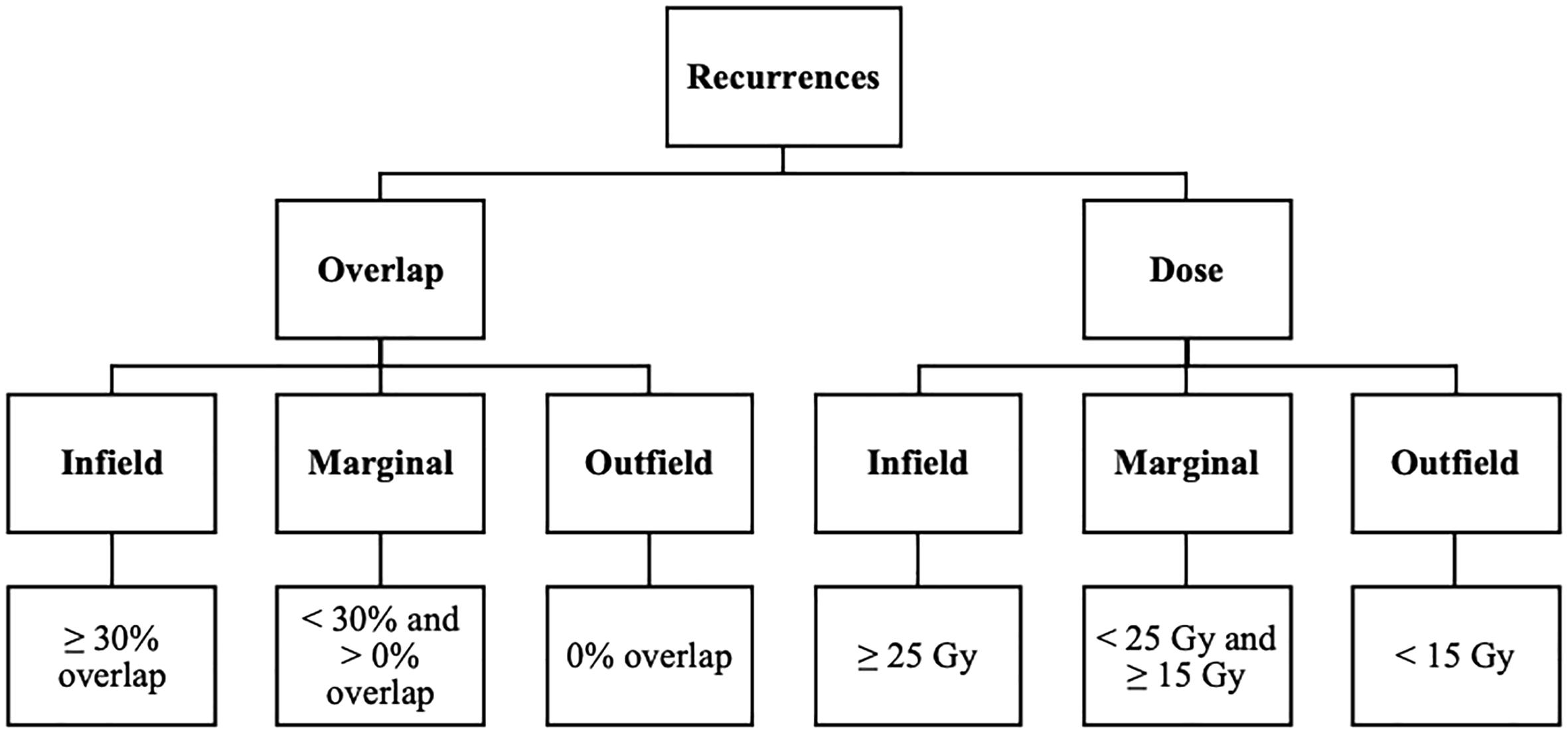

Supplement: Supplementary Figure 1 [file mmc1.jpg]
