# Supplementary material for: Patterns of prostate recurrence after focal salvage prostate brachytherapy for radiorecurrent prostate cancer
Source: Clin Transl Radiat Oncol. 2025 Sep 4;56:101043. doi: 10.1016/j.ctro.2025.101043 (PMC12797306; doi:10.1016/j.ctro.2025.101043)
Supplement: Supplementary Data 2 [file mmc2.docx]

Supplementary figure 1. Classification of recurrences based on overlap and dose definition.
